# Supplementary material for: Effect of Percutaneous Electric Stimulation with High-Frequency Alternating Currents on the Sensory-Motor System of Healthy Volunteers: A Double-Blind Randomized Controlled Study
Source: J Clin Med. 2022 Mar 25;11(7):1832. doi: 10.3390/jcm11071832 (PMC8999650; doi:10.3390/jcm11071832)
Supplement: Supplementary file 1 [file jcm-11-01832-s001.zip › jcm-1638242-supplementary.pdf]

Table S1. Descriptive values. Abbreviations; PRE: Pre-intervention, DUR: During the intervention. NA: Not applicable. SNAP: sensitive nerve action potential. SD: Standard Deviation.

Supplementary Data

	Sham group				10 kHz group				20 kHz group			
**Outcomes** % Mean (SD)	Pre	Dur	Post 0	Post 15	Pre	Dur	Post 0	Post 15	Pre	Dur	Post 0	Post 15
Strength	8.2 (1.9)	NA	8.1 (2.1)	7.9 (2.3)	9.9 (2.9)	NA	9.0 (2.6)	8.9 (2.8)	8.9 (2.3)	NA	7.8 (2.1)	7.8 (2.0)
Myotonometry Frequency	20.2 (1.8)	20.6 (2.0)	20.3 (2.5)	20.6 (2.2)	19.8 (1.7)	21.1 (2.1)	20.5 (1.9)	20.4 (1.6)	19.9 (2.1)	20.7 (2.0)	21.3 (2.4)	21.2 (2.2)
Myotonometry Decrement	1.7 (0.3)	1.7 (0.3)	1.7 (0.3)	1.8 (0.3)	1.6 (0.2)	1.7 (0.3)	1.7 (0.3)	1.7 (0.3)	1.6 (0.3)	1.7 (0.3)	1.7 (0.3)	1.7 (0.3)
Myotonometry Stiffness	350.1 (43.4)	366.4 (49.6)	359.3 (52.9)	358.8 (47.4)	353.1 (49.9)	390.9 (53.7)	375.3 (52.8)	361.5 (44.9)	354.3 (41.5)	370.9 (38.7)	384.3 (48.3)	382.7 (45.0)
Pain Pressure Threshold	53.8 (14.9)	50.6 (16.2)	50.5 (14.5)	49.8 (17.3)	58.4 (26.4)	53.7 (24.7)	58.0 (26.0)	56.8 (22.9)	62.2 (18.5)	59.3 (22.8)	58.7 (22.1)	59.5 (24.0)
Amplitude SNAP	0.19 (0.01)	NA	0.02 (0.02)	0.02 (0.01)	0.02 (0.02)	NA	0.03 (0.03)	0.03 (0.03)	0.02 (0.01)	NA	0.03 (0.03)	0.02 (0.01)
Nerve Conduction Velocity	47.0 (4.6)	NA	44.4 (3.8)	44.1 (3.8)	45.4 (11.7)	NA	45.3 (7.2)	45.2 (10.3)	47.3 (5.8)	NA	46.3 (7.6)	44.7 (6.2)
Forearm Temperature	28.7 (1.2)	28.9 (1.3)	28.9 (1.3)	28.9 (1.4)	29.5 (1.2)	29.1 (2.0)	29.1 (1.8)	28.9 (2.4)	28.8 (1.4)	29.2 (2.1)	29.1 (1.8)	29.0 (1.9)
Hand Temperature	28.5 (2.0)	27.8 (3.2)	27.7 (2.8)	27.3 (3.1)	28.4 (2.7)	27.5 (3.8)	27.8 (3.1)	27.5 (3.1)	28.3 (2.0)	28.0 (2.9)	27.7 (2.2)	27.2 (2.5)
